# Physiological and molecular characterization of aluminum resistance in *Medicago truncatula*

**DOI:** 10.1186/1471-2229-8-89

**Published:** 2008-08-19

**Authors:** Divya Chandran, Natasha Sharopova, Kathryn A VandenBosch, David F Garvin, Deborah A Samac

**Affiliations:** 1 Department of Plant Biology, University of Minnesota, 250 Biological Sciences Center, St. Paul, MN 55108, USA; 2 USDA-ARS-Plant Science Research, St. Paul, MN 55108, USA; 3 Center for Microbial and Plant Genomics, University of Minnesota, St. Paul, MN 55108, USA; 4 Department of Agronomy and Plant Genetics, University of Minnesota, 411 Borlaug Hall St. Paul, MN 55108, USA; 5 Department of Plant Pathology, University of Minnesota, 495 Borlaug Hall, St. Paul, MN 55108, USA

## Abstract

**Background:**

Aluminum (Al) toxicity is an important factor limiting crop production on acid soils. However, little is known about the mechanisms by which legumes respond to and resist Al stress. To explore the mechanisms of Al toxicity and resistance in legumes, we compared the impact of Al stress in Al-resistant and Al-sensitive lines of the model legume, *Medicago truncatula *Gaertn.

**Results:**

A screen for Al resistance in 54 *M. truncatula *accessions identified eight Al-resistant and eight Al-sensitive lines. Comparisons of hydroponic root growth and root tip hematoxylin staining in an Al-resistant line, T32, and an Al-sensitive line, S70, provided evidence that an inducible Al exclusion mechanism occurs in T32. Transcriptional events associated with the Al resistance response were analyzed in T32 and S70 after 12 and 48 h Al treatment using oligonucleotide microarrays. Fewer genes were differentially regulated in response to Al in T32 compared to S70. Expression patterns of oxidative stress-related genes, stress-response genes and microscopic examination of Al-treated root tips suggested a lower degree of Al-induced oxidative damage to T32 root tips compared to S70. Furthermore, genes associated with cell death, senescence, and cell wall degradation were induced in both lines after 12 h of Al treatment but preferentially in S70 after 48 h of Al treatment. A multidrug and toxin efflux (MATE) transporter, previously shown to exude citrate in *Arabidopsis*, showed differential expression patterns in T32 and S70.

**Conclusion:**

Our results identified novel genes induced by Al in Al-resistant and sensitive *M. truncatula *lines. In T32, transcription levels of genes related to oxidative stress were consistent with reactive oxygen species production, which would be sufficient to initiate cell death of Al-accumulating cells thereby contributing to Al exclusion and root growth recovery. In contrast, transcriptional levels of oxidative stress-related genes were consistent with excessive reactive oxygen species accumulation in S70 potentially resulting in necrosis and irreversible root growth inhibition. In addition, a citrate-exuding MATE transporter could function in Al exclusion and/or internal detoxification in T32 based on Al-induced transcript localization studies. Together, our findings indicate that multiple responses likely contribute to Al resistance in *M. truncatula*.

## Background

Aluminum (Al) toxicity is one of the important factors limiting crop productivity in acid soils (pH<5.0) [1], which occupy approximately 30% of the world's arable land [2]. Under acidic conditions, Al(H_2_O)_6_^3+ ^(Al^3+^) is released into the soil solution at levels that inhibit plant root growth and impair water and mineral uptake [3]. Despite decades of research on Al resistance, little is known about the mechanisms by which legumes respond to and tolerate Al stress. The model legume, *Medicago truncatula *Gaertn., which is a close relative of alfalfa, has a relatively small diploid genome, short generation time and prolific seed production [4] and therefore serves as an ideal model system to study Al toxicity and resistance mechanisms in legumes.

The root apex is considered to be the primary target of Al toxicity. Exposure of the root apex to Al results in a rapid inhibition of root growth [5]. Al disrupts root cell expansion and elongation, prior to inhibiting cell division [6] and interferes with a wide range of physical and cellular processes. Inhibition of root growth may occur as a result of Al-induced decrease in cell wall extensibility [7], callose formation [8], inhibition of H^+^-ATPase activity [9], disruption of calcium homeostasis [10], stabilization of cortical cell microtubules [11] and/or alteration in chromatin structure by DNA binding [12].

Many plant species exhibit significant genetic variability in their ability to resist and tolerate Al toxicity. *M. truncatula *exhibits a natural variation in tolerance to low pH [13] and Al toxicity [14]. Current models for Al resistance mechanisms include exclusion of Al from the root apex and internal detoxification of Al transported into the root symplasm [6]. Al-induced secretion of organic acid (OA)-chelators is considered to be the primary mechanism of Al exclusion from the root apex. Also, chelation of Al by OAs within the root symplasm has been observed in some plant species [15]. A number of studies have indicated that OA chelation may not be the only mechanism responsible for Al resistance [16-19].

Over the last decade, researchers have debated whether the induced expression of genes or the activation of pre-formed proteins or both are necessary to combat Al toxicity. The biochemical machinery for root Al exclusion via organic acid release appears to be in place before exposure to Al in some species [20,21]. In other species a delay in secretion is observed, indicating that gene induction may be required [22-24]. Several studies have identified genes that are up-regulated under Al stress conditions. However, most of these genes were considered to be general stress response genes since they were induced in response to other stresses (other metal toxicities, low Ca, wounding and oxidative stress) and to similar levels in both Al-resistant and Al-sensitive genotypes [25-30].

In this report, we identified Al-resistant and Al-sensitive *M. truncatula *lines and quantified differences in Al effects on root physiology and gene expression between these lines. Based on our findings we propose that multiple responses including Al exclusion by Al-induced cell death of Al-accumulating cells and organic acid efflux and internal detoxification by OA chelation may contribute towards higher Al resistance in *M. truncatula*.

## Results and Discussion

### Identification of Al-resistant and Al-sensitive *M. truncatula*

Plants from 54 *M. truncatula *accessions were screened using a hydroponic assay with 2.5 μM Al concentration. At this Al concentration, the relative root growth (RRG) of the reference *M. truncatula *genotype A17 was 33%. In a previously published study [31], 0.1 N KOH was used to adjust the pH of the Al solutions to 4.5. The addition of base can result in the hydrolysis of Al(H_2_O)_6_^3+ ^(Al^3+^) into monomeric species such as Al(H_2_O)_5_OH^2+ ^[32]. This could have potentially lowered the concentration of the toxic form of Al (Al^3+^) in the solution. In the current study, we avoided the use of base. Therefore, similar levels of root growth inhibition as obtained previously were achieved by using 10-fold lower Al concentrations in the current study.

The RRG values of the different lines were normalized to that of A17 to rank them according to their level of resistance. Eight lines with normalized values greater than 2.0 were considered Al-resistant and eight lines with values below 0.5 as Al-sensitive (Table 1). No correlation was observed between seed size and root growth (data not shown). An Al-resistant line generated from a single seed of the accession PI 384662 from Morocco (designated T32), and an Al-sensitive line similarly derived from the Italian accession PI577613 (designated S70), were selected to study physiological and molecular aspects of Al resistance. T32 showed the highest RRG in Al solution and was the obvious choice for the resistant line. Although S70 was not the most Al-sensitive line in the screen, it was selected because plant to plant variation in root growth in nutrient solution was minimal compared to other Al-sensitive lines and root growth rates of S70 were most similar to that of T32 in control solutions (72 h root growth of T32 in control solutions was 13 mm while that of S70 was 15.8 mm).

**Table 1:** Mean relative root growth (n = 6) of *M. truncatula *lines after 2 d of growth in 2.5 μM Al solution.

Line Designation	Accession Number	Country of Origin	Relative root growth (RRG)	(±)SE	*P-*value (*<0.05 **<0.01)	RRG (normalized to A17)
USDA Collection						
A32 (T32)	PI 384662	Morocco	0.93	0.05	0.001 **	2.92
A43	PI 577628	Spain	0.91	0.09	0.000 **	2.85
A41	PI 319045	Spain	0.88	0.05	0.000 **	2.75
A84	W6 6050	Tunisia	0.88	0.17	0.000 **	2.74
A10	PI 197361	Australia	0.81	0.11	0.000 **	2.52
A62	PI 464815	Turkey	0.80	0.28	0.001 **	2.50
A47	W6 6099	Portugal	0.71	0.09	0.000 **	2.20
A36	PI 516937	Morocco	0.67	0.13	0.000 **	2.08
A85	W6 6110	Italy	0.63	0.11	0.000 **	1.97
A55	W6 6000	France	0.60	0.07	0.000 **	1.88
A34	PI 516927	Morocco	0.60	0.08	0.000 **	1.87
A77	W6 6012	Italy	0.59	0.09	0.001 *	1.85
A91	PI 577617	Greece	0.59	0.03	0.000 **	1.84
A65	PI 577434	Tunisia	0.58	0.08	0.001 **	1.81
A100	W6 5983	Cyprus	0.56	0.10	0.003 **	1.75
A50	PI 577608	France	0.55	0.15	0.018 *	1.70
A11	PI 243884	Australia	0.54	0.12	0.012 *	1.69
A28	PI 384636	Morocco	0.52	0.06	0.005 **	1.63
A12	PI 442892	Australia	0.50	0.09	0.020 *	1.56
A88	PI 577599	Greece	0.49	0.13	0.052	1.54
A3	PI 190084	Australia	0.49	0.09	0.028 *	1.53
A87	PI 566887	Greece	0.48	0.21	0.183	1.49
A89	PI 577601	Greece	0.47	0.12	0.087	1.46
A101	W6 5984	Cyprus	0.44	0.06	0.083	1.38
A21	PI 577641	Australia	0.42	0.10	0.210	1.31
A15	PI 469099	Australia	0.38	0.07	0.438	1.19
A40	PI 244285	Spain	0.38	0.07	0.427	1.18
A66	PI 577619	Tunisia	0.35	0.15	0.787	1.10
A5	PI 190089	Australia	0.34	0.05	0.852	1.06
	A17	Australia	0.33	0.03	NA	1.00
A92	PI 577618	Greece	0.31	0.11	0.818	0.97
A93	PI 577604	Cyprus	0.30	0.07	0.690	0.94
A18	PI 517257	Australia	0.28	0.16	0.633	0.88
A82	W6 6047	Tunisia	0.28	0.03	0.418	0.87
A17	PI 469102	Australia	0.27	0.05	0.374	0.85
A42	PI 319051	Spain	0.23	0.08	0.000 **	0.72
A76	W6 5964	Italy	0.23	0.04	0.112	0.71
A19	PI 566888	Australia	0.19	0.07	0.044 *	0.60
A90	PI 577602	Greece	0.17	0.08	0.026 *	0.53
A70 (S70)	PI 577613	Italy	0.15	0.02	0.002 **	0.48
A60	PI 577614	Malta	0.15	0.10	0.028 *	0.48
A7	PI 190091	Australia	0.15	0.09	0.018 *	0.48
A31	PI 384660	Morocco	0.15	0.04	0.012 *	0.47
A4	PI 190087	Australia	0.14	0.04	0.004 **	0.44
A56	PI 493295	Portugal	0.13	0.07	0.005 **	0.40
A48	PI 535739	Libya	0.12	0.05	0.002 **	0.39
A79	W6 6025	Italy	0.08	0.06	0.001 **	0.26
						
French Collection						
	DZA45.5	Algeria	0.61	0.08	0.000 **	1.91
	TN8.3	Tunisia	0.41	0.04	0.132	1.28
	TN1.11	Tunisia	0.35	0.03	0.656	1.10
	F83005.9	France	0.33	0.14	0.941	1.04
	F8005.5	France	0.30	0.03	0.586	0.93
	A20	Australia	0.25	0.05	0.207	0.80
	DZA315.16	Algeria	0.24	0.03	0.099	0.74
	TN6.18	Tunisia	0.19	0.11	0.124	0.59

RRG, Ratio of root growth in 2.5 μM Al solution to root growth in 0 μM Al solution; SE, standard error of the mean; *P-*value, significance comparing mean relative root growth for each line with that of A17. Student's t-test (assuming equal variance) using a two-tailed distribution was employed for the statistical test.

### Aluminum dose-response root growth

**Figure 1:**
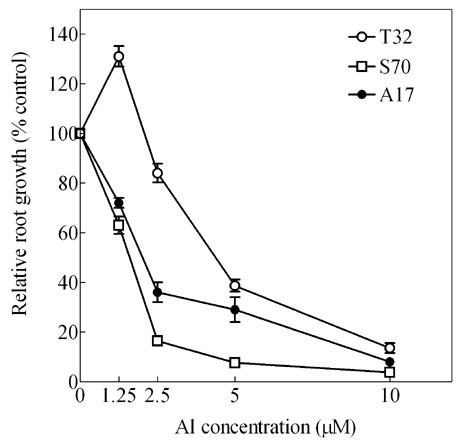
**Effect of Al-dose on root growth in *M. truncatula*.** RRG ± SE of T32, A17 and S70 seedlings grown at 1.25, 2.5, 5 and 10 μM Al for 48 h.

Relative root growth of A17, T32, and S70 decreased in an Al dose-dependent manner over 48 h of Al exposure (Figure 1). However, differences in Al resistance between T32 and S70 were observed at all Al concentrations evaluated. At lower solution Al concentrations (1.25 and 2.5 μM), T32 exhibited the highest RRG values (120% and 90%, respectively), followed by A17 (70% and 35%, respectively), and S70 (65% and 15%, respectively). The maximum difference in RRG between T32 and S70 was observed at 2.5 μM solution Al concentration.

**Figure 2:**
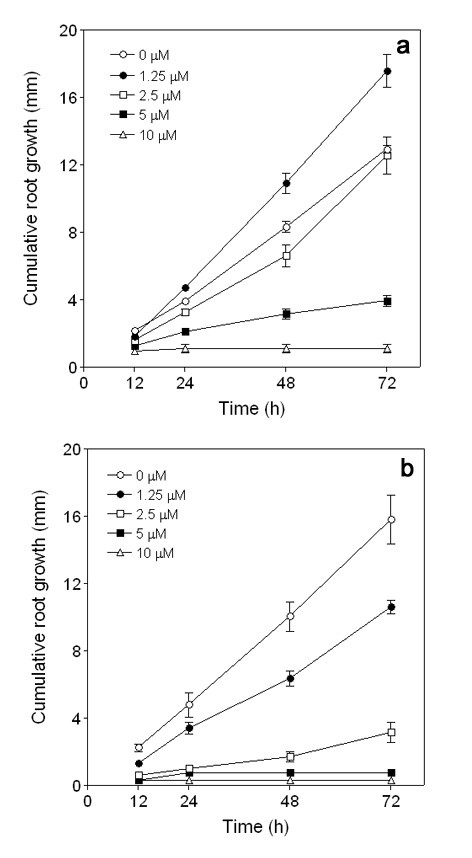
**Effect of Al-dose and time on root growth of *M. truncatula*.** Cumulative root growth of seedlings exposed to 0, 1.25, 2.5, 5 and 10 μM Al for 12, 24, 48 and 72 h. Data represent the mean root growth values ± SE of eight seedlings from three independent experiments. (a) T32 seedlings. (b) S70 seedlings.

Within the first 24 h of Al treatment, root growth inhibition in T32 occurred at all Al concentrations tested except at 1.25 μM Al (Figure 2a). In 1.25 μM solution Al concentration the root growth of T32 seedlings was approximately 20% higher than the control seedlings after 24 h. This phenomenon has been observed in wheat, and it was suggested that enhanced root growth might be the result of alleviation of H^+ ^stress under acidic conditions by Al [33]. Alternatively, addition of Al to the nutrient solution may influence the bioavailability of other ions in a manner that stimulates growth [34]. Interestingly, it has been shown that the low concentration Al-induced increase in root growth of *Quercus serrata *was not caused by the amelioration of H^+ ^toxicity by Al [35]. In contrast, root growth inhibition occurred at all Al concentrations in S70 at 24 h (Figure 2b). By 72 h, recovery of root growth to control rates was observed in T32 at 2.5 μM Al. Depletion of Al from the nutrient solution is not a likely explanation for this result given the large solution volume and small root mass involved. In S70, no recovery was observed and root growth inhibition appeared to be constant throughout the time course of the experiment. The stimulation of root growth at 1.25 μM Al and recovery of root growth at 2.5 μM Al suggests that the Al resistance of T32 is inducible. In an Al-resistant maize cultivar, there was a lag time of more than 4 h before the root tips were efficiently protected against Al toxicity [36]. Similarly, in 2.5 μM Al solutions, a lag period between Al stress perception and functional expression of induced Al resistance would explain the observed root growth inhibition and recovery pattern in T32.

### Al accumulation in T32 and S70 root tips

**Figure 3:**
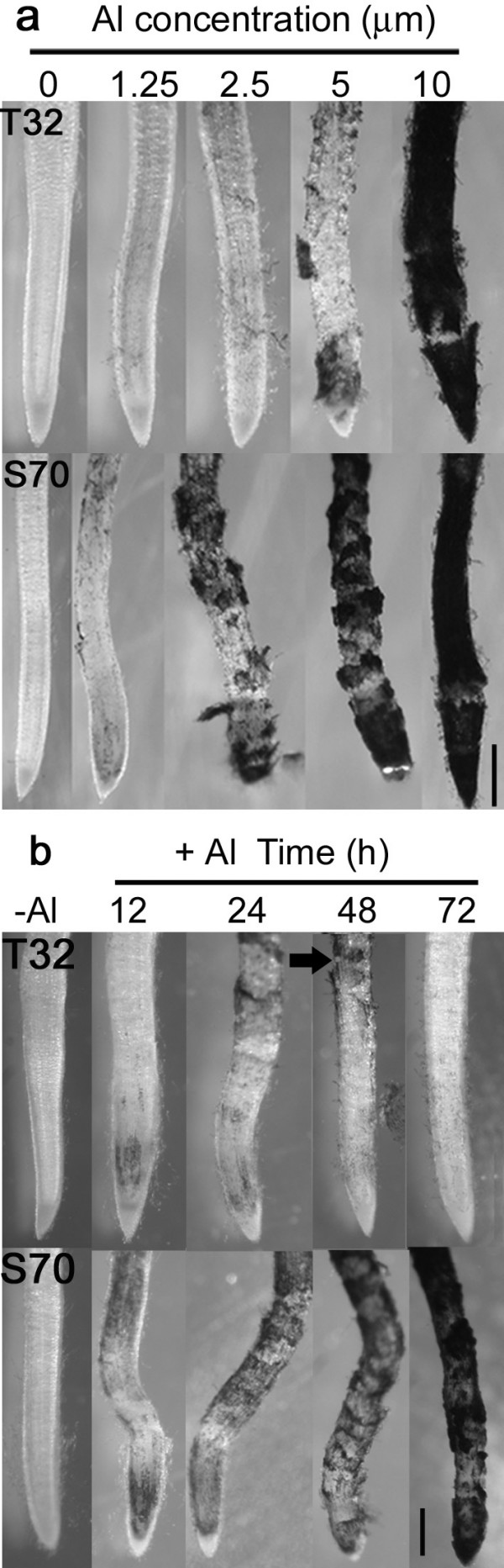
**Al-accumulation in *M. truncatula *root apices.** (a) Hematoxylin staining of T32 and S70 seedlings exposed to a range of Al concentrations for 48 h. (b) Hematoxylin staining of root apices of *M. truncatula *grown in 2.5 μM Al solution for 0 to 72 h. Scale bar represents 500 μm.

The degree of hematoxylin staining in root tips provides a semi-quantitative measure of Al content, and is inversely proportional to both the ability of a genotype to exclude Al from the root apex, and its Al resistance [37]. The greatest differentiation between T32 and S70 was observed at 2.5 μM Al, at which root tips of T32 exhibited minimal staining and root tips of S70 were more intensely stained (Figure 3a). Similar differences were observed at 5 μM Al. However, at 10 μM Al, root tips of both lines were intensely stained, indicating an inability to effectively exclude Al at such high concentrations.

Roots of T32 and S70 were stained with hematoxylin following different lengths of exposure to 2.5 μM Al to determine whether the recovery in root growth observed in T32 was a consequence of a decrease in Al accumulation in the root tips. Within 12 h of Al treatment, root tips of both lines were lightly stained (Figure 3b). By 72 h, no staining was observed in T32. In contrast, root tips of S70 were intensely stained. The increased Al resistance of T32 may depend on an induced ability to exclude Al, since no visible hematoxylin staining was observed in T32 root tips at 72 h. If Al exclusion is inducible in T32, the Al that was initially taken up by the roots would still be present, and would likely be visible by hematoxylin staining. Figure 3b shows visible hematoxylin staining approximately 3 mm from the root tip at 48 h of Al treated roots (black arrow), demonstrating that Al had accumulated in root cells prior to root growth recovery. In contrast, S70 root tips showed increased Al accumulation coupled with severe cell damage that extended 1 to 2 mm behind the root tip (Figure 3b). This pattern of injury is similar to previous observations in Al-sensitive maize root apices [38]. The hematoxylin staining result is concordant with the root growth data, indicating that greater Al accumulation is correlated with greater root growth inhibition in *M. truncatula*.

### Differential expression of genes in response to Al

**Figure 4:**
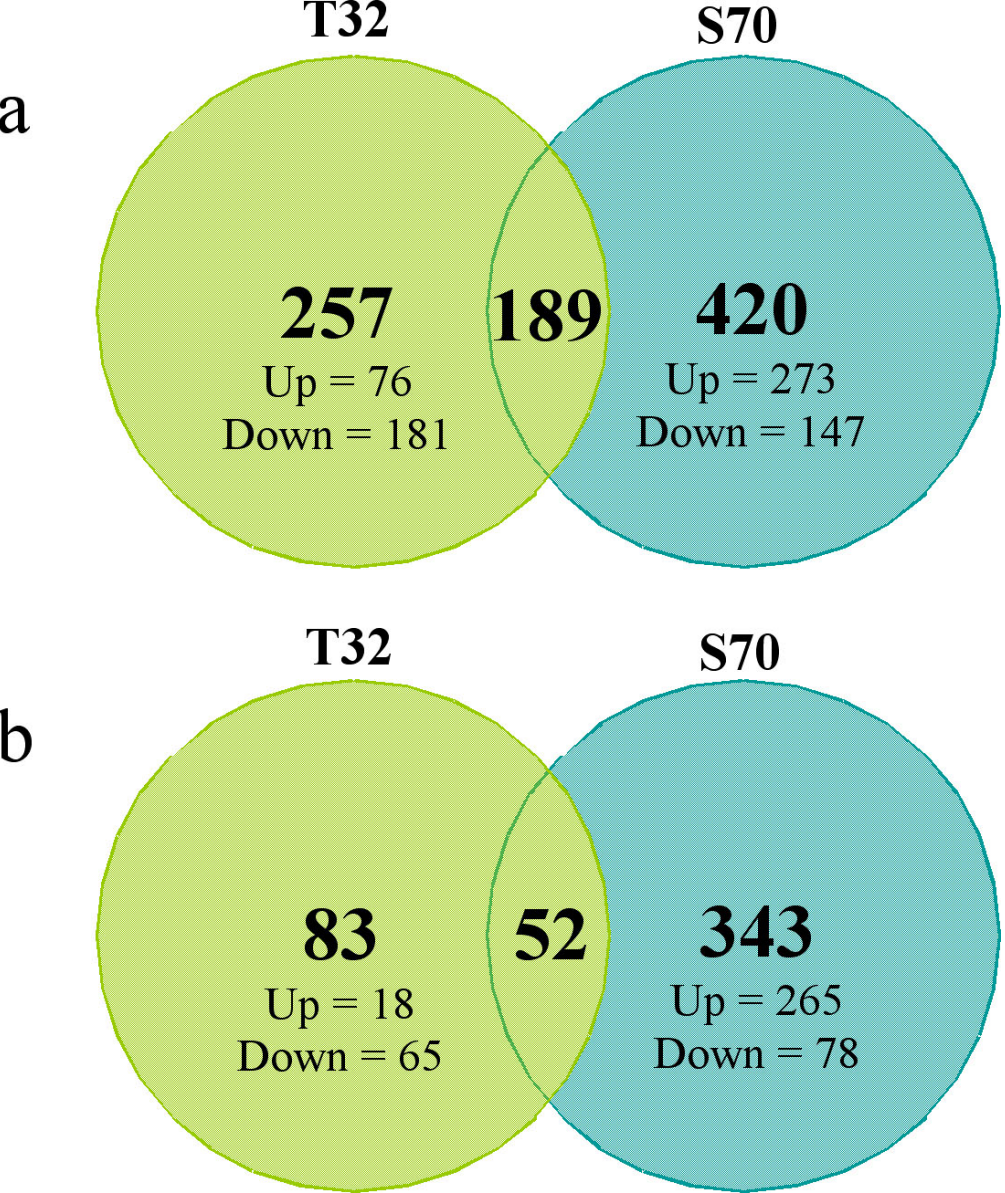
**Genes with significantly altered expression in Al-treated root tips compared to control root tips in T32 and S70.** (a) 12 h Al treatment. (b) 48 h Al treatment.

The molecular responses underlying differences in Al resistance were investigated using the *M. truncatula *AROS (version 1.0) arrays (Operon Biotechnologies Inc., Huntsville, AL) consisting of probes for approximately 16,000 *M. truncatula *expressed genes. Al-induced gene expression was compared in T32 and S70 after 12 and 48 h of +/- 2.5 μM Al treatment. We selected 12 h since root growth inhibition was observed at that time point in T32. We selected the 48 h time point since root growth rates recovered to that of the controls by 72 h in T32. These time points were chosen to identify transcriptional differences associated with Al-toxicity and resistance responses between the two lines. To detect significant genes with Al-regulated expression and to eliminate those that have inconsistent expression data among replicated experiments, we employed a statistical method adapted specifically for microarrays, which allows estimation of the false discovery rate (FDR) for multiple testing [39]. A delta criterion that allowed a FDR < 0.5% was applied. Genes that satisfied the statistical threshold were identified as significantly up- or down-regulated in Al-treated roots. In addition, we used a 2-fold change cut-off for the significant genes. Normalized and raw data have been submitted to NCBI Gene Expression Omnibus (Accession No. GSE6946). In both lines, the expression of a majority of transcripts appeared unchanged at both time points with Al treatment. As shown in Figure 4, more genes were significantly altered by ≥ 2.0-fold in 12 h Al-treated root tips compared to 48 h Al-treated root tips. Additionally, at both time points, a greater number of Al-induced genes were observed in S70 root tips (12 h = 365; 48 h = 287). Since root growth of S70 was inversely correlated with Al accumulation at these time points, the greater number of up-regulated genes in S70 at both time points likely corresponds to greater Al stress perception and may reflect Al toxicity responses. In contrast, the numbers of down-regulated genes were similar in both lines and at both time points (Figure 4). It has been suggested that adaptive reprogramming to stressful conditions may require a higher number of down-regulated genes [40]; therefore, the similar numbers of down-regulated genes at both time points in both lines might reflect an overlap in Al stress adaptation responses.

The genes showing significantly different transcript accumulation patterns in response to Al in T32 and S70 experiments were further compared to determine the number that were up- or down-regulated in response to Al treatment by 2-fold or more in both lines vs. only in one line (Figure 4). At the 12 h time point, 257 genes were uniquely expressed in T32 and 420 in S70. By 48 h T32 had 83 uniquely expressed genes and 343 were expressed only in S70. These differences may represent differences in Al stress perception and adaptation in T32 and S70 root tips. The large number of shared genes at the 12 h time point (189) might reflect an overlap in the Al toxicity responses in both lines. A list of selected significant differentially regulated genes is presented in Table 2. Functional categorization for all genes was based on Gene Ontology (GO) process information .

**Table 2:** Selected genes differentially regulated by ≥ 2.0-fold or ≤ 0.5-fold in at least one *M. truncatula *line in response to Al treatment.

TIGR TC	Tentative Annotation	GO process iD	Fold Change T32		Fold Change S70	
	**Cell Wall Modification**		12 h	48 h	12 h	48 h
			
TC96658	Xyloglucan endotransglucosylase putative	Carbohydrate metabolism GO:0005975	2.2	2.9	5.3	10.7
TC100486	Xyloglucan endotransglycosylase putative	Carbohydrate metabolism	2.3	0.9	1.3	0.8
TC95073	Polygalacturonase like protein	Carbohydrate metabolism	2.6	0.6	4.3	1.3
TC96355	Polygalacturonase	Carbohydrate metabolism	1.1	2.3	ND	4.1
TC111920	Polygalacturonase PG1 putative	Carbohydrate metabolism	1.4	2.8	1.5	3.7
TC110038	Pectinesterase putative	Cell wall modification GO:0042545	2.0	1.1	1.3	0.7
TC94920	Probable pectinesterase precursor	Cell wall modification	2.2	0.8	4.5	ND
TC107655	Xyloglucanase inhibitor putative	Unknown	1.3	1.3	4.4	ND
TC94309	Xyloglucanase inhibitor putative	Unknown	1.1	0.9	2.0	0.9
TC94310	Xyloglucanase inhibitor putative	Unknown	1.1	0.8	2.1	0.9
TC98964	Caffeic acid O-methyltransferase	Lignin biosynthesis GO:0009809	1.3	0.9	1.5	2.0
TC107848	Caffeic acid O-methyltransferase II	Lignin biosynthesis	1.2	1.2	2.1	1.3
TC100394	Caffeic acid O-methyltransferase	Lignin biosynthesis	1.1	ND	2.1	2.8
TC94484	Expansin	Cell expansion GO:0009831	1.5	1.5	2.0	3.7
TC109283	Expansin	Cell expansion	1.4	1.5	1.4	3.0
TC100685	Pectate lyase	Unknown	1.4	1.8	1.2	4.7
TC96079	Pectate lyase	Unknown	1.4	1.8	1.9	4.6
TC108882	β-1,4-glucanase	Carbohydrate metabolism	1.3	1.5	ND	10.6
TC108539	Arabinogalactan protein-like	Cell adhesion GO:0007155	0.6	1.1	1.0^ns^	3.8
TC94068	Fasciclin-like AGP 14	Cell adhesion	ND	1.1	1.0	2.3
TC108519	Fasciclin-like AGP 14	Cell adhesion	0.6	1.5	ND	6.9
TC104866	Pectinesterase like protein	Cell wall modification	0.5	1.3	0.3	0.5
						
	**Oxidative Stress-ROS Generation**					
TC95154	Peroxidase	Response to oxidative stress GO:0006979	6.3	3.9	16.4	3.0
TC103581	Peroxidase	Response to oxidative stress	5.2	2.5	9.9	2.6
TC108789	Peroxidase 11 precursor	Response to oxidative stress	4.2	3.5	7.6	2.4
TC97623	Peroxidase	Response to oxidative stress	2.6	3.1	ND	0.7
TC107670	Peroxidase	Response to oxidative stress	3.0	1.6	4.3	1.3
TC108447	Peroxidase 2	Response to oxidative stress	2.9	0.4	2.3	0.8
TC95164	Peroxidase 55 precursor	Response to oxidative stress	2.0	0.9	3.2	1.9
TC111928	Peroxidase precursor	Response to oxidative stress	1.6	2.9	2.4	2.8
TC103214	Peroxidase	Response to oxidative stress	1.4	0.7	1.4	2.4
TC101009	Peroxidase	Response to oxidative stress	1.6	0.9	ND	2.8
TC108315	Cationic peroxidase 2 precursor	Response to oxidative stress	1.1	0.9	1.9	2.7
TC111871	Peroxidase	Response to oxidative stress	1.4	1.4	2.4	2.3
TC94676	Peroxidase precursor	Response to oxidative stress	1.2	1.2	2.2	2.5
TC108988	Germin-like protein precursor	Response to oxidative stress	2.9	ND	1.6	0.5
TC100563	Germin-like protein	Response to oxidative stress	1.6	ND	2.3	1.1
TC94265	Germin-like protein	Response to oxidative stress	0.9^ns^	0.6	2.1	1.9^ns^
TC107416	Carbohydrate oxidase	Electron transport GO:0006118	1.2	1.4	4.3	1.7
TC103024	Peroxidase	Response to oxidative stress	0.4	0.3	0.4	0.1
TC100904	Peroxidase precursor	Response to oxidative stress	0.2	0.2	0.1	0.2
TC102226	Peroxidase	Response to oxidative stress	0.3	ND	0.1	0.2
TC104806	Probable peroxidase	Response to oxidative stress	0.4	0.3	1.5	0.4
TC106851	Peroxidase 2	Response to oxidative stress	0.4	0.5	0.6	0.5
TC108234	Peroxidase precursor	Response to oxidative stress	0.2	0.3	0.2	0.7
TC96817	Germin-like protein	Response to oxidative stress	0.5^ns^	1.4	0.3	0.8
TC100175	Lipoxygenase	Jasmonic acid biosynthesis GO:0009695	0.4	0.4	ND	1.1
TC100188	Lipoxygenase LoxN2	Jasmonic acid biosynthesis	0.7	0.5	0.5	0.9
TC100155	Lipoxygenase	Jasmonic acid biosynthesis	0.6	0.3	0.5	0.7
TC100171	Lipoxygenase	Jasmonic acid biosynthesis	0.6	0.3	0.5	0.8
						
	**Oxidative Stress- ROS Scavenging**					
TC94929	Quinone-oxidoreductase QR1	Threonine catabolism GO:0006567	2.0	1.2	1.8	0.9^ns^
TC110730	Quinone-oxidoreductase QR1	Threonine catabolism	2.2	1.3	1.3	0.8
TC107874	NADH-ubiquinone oxidoreductase	Electron transport	1.1^ns^	2.3	0.3	0.8
TC95262	Blue copper protein precursor	Electron transport	3.1	1.6	1.9	1.3^ns^
TC103046	Thioredoxin-like 4	Electron transport	2.1	1.3	5.3	1.8
TC96215	Thioredoxin H2	Electron transport	0.6	2.4	0.4	0.7
TC104708	Thioredoxin H2	Electron transport	ND	2.4	1.3^ns^	3.0
TC104047	Thioredoxin 3	Electron transport	0.9	2.3	0.8	3.8
BM813626	Ascorbate peroxidase	Response to oxidative stress	0.7	0.9	1.6	2.6
TC101862	Alternative oxidase 3	Alternative respiration GO:0010230	1.3	0.8	1.7	2.0
TC95582	Tyrosine aminotransferase	Vitamin E biosynthesis GO:0010189	1.5	2.5	8.3	ND
TC100815	4-hydroxyphenylpyruvate dioxygenase	Vitamin E biosynthesis	1.2	1.1	2.3	1.3
TC108214	Glutathione S-transferase GST 5	Response to oxidative stress	2.2	ND	1.4	1.4
TC100556	Glutathione-S-transferase	Response to oxidative stress	2.2	1.1	2.3	ND
TC105598	Probable glutathione S-transferase	Response to oxidative stress	1.3	1.4	2.3	1.0^ns^
TC95231	Probable glutathione S-transferase	Response to oxidative stress	1.3	1.0	2.1	0.8
TC106943	Glutathione S-transferase GST 8	Response to oxidative stress	1.3	1.0^ns^	2.1	ND
TC94362	Glutathione S-transferase GST 8	Response to oxidative stress	1.2	1.2	2.0	1.0
TC106973	Glutathione S-transferase	Response to oxidative stress	1.1	1.3	3.3	ND
TC95247	Glutathione S-transferase GST 14	Response to oxidative stress	1.0^ns^	0.7	2.2	0.2
TC95380	Glutathione S-transferase GST 15	Response to oxidative stress	ND	1.2	0.5	0.8
TC108817	Glutathione S-transferase GST 11	Response to oxidative stress	ND	1.7	0.7	0.4
						
	**Pathogenesis-Related (PR) Proteins**					
TC93997	PR-protein PR-4	Defense response GO:0006952	2.6	1.7	5.0	2.1
BQ138448	PR- protein	Defense response	ND	1.3	2.3	3.2
TC101688	PR-protein 4A	Defense response	0.6	1.0^ns^	2.7	1.4
TC94004	PR-protein 4A	Defense response	0.6	0.9	2.7	1.6
TC94274	Thaumatin-like protein PR-5b precursor	Response to pathogen GO:0042828	1.3	1.0	2.1	1.8
TC107543	Thaumatin-like protein	Response to pathogen	1.6	1.1	2.7	1.9
TC96745	PR-protein homolog	Defense response	1.5	1.2	1.6	2.4
TC102065	β-1,3-glucanase (putative)	Carbohydrate metabolism	2.4	0.5	2.3	3.0
TC96253	β-1,3-glucanase like protein	Carbohydrate metabolism	2.3	1.3	2.0	0.9
						
	**Isoflavonoid Biosynthesis**					
TC94931	Cytochrome P450	Electron transport	2.5	1.4	2.2	1.6^ns^
TC101508	Isoflavone 2'-hydroxylase	Electron transport	1.6	1.0^ns^	2.6	1.5
TC95424	Isoflavone 3'-hydroxylase	Electron transport	1.8	0.9	3.8	ND
TC96039	Isoflavone reductase related protein	Response to oxidative stress	1.8	0.9	3.1	1.2
TC106939	Isoflavone synthase putative	Electron transport	1.3	ND	2.2	1.0^ns^
TC97999	Cytochrome P450 monooxygenase	Electron transport	1.2	0.9	2.1	1.4
						
	**Stress Response**					
TC108137	ABA-responsive protein-like	Response to desiccation GO:0009269	1.7	1.4	2.7	1.3
TC108387	Abscisic acid-responsive protein	Response to desiccation	1.8	1.2	2.5	2.0
TC106508	Abscisic stress ripening protein homolog	Response to desiccation	1.3	1.3	2.6	2.7
BG454018	Late embryogenesis abundant protein	Response to desiccation	5.5	0.8^ns^	12.9	1.2
TC94387	Late embryogenesis abundant protein 2	Response to desiccation	1.5	1.1	4.2	0.9
TC94389	Late embryogenesis abundant protein 2	Response to desiccation	1.8	1.2	5.1	0.9
TC94508	Seed maturation protein LEA 4	Response to desiccation	1.9	1.0	4.6	0.9
TC101519	Patatin-like protein 1	Lipid metabolism GO:0006629	3.6	0.7	6.6	0.5
TC101298	Glucosyl transferase	Metabolism GO:0008152	0.5	0.6	2.0	ND
TC94863	Glucosyltransferase putative	Metabolism	1.6	0.8	2.3	ND
TC100689	Glucosyltransferase-6	Metabolism	1.6	1.5	2.0	1.5
TC110504	Glucosyltransferase-7	Metabolism	1.4	1.4	6.3	ND
TC103147	Tumor-related protein	Unknown	0.5	0.7	2.3	4.2
TC107043	ER6 protein (universal stress protein)	Response to stress GO:0006950	0.3	1.2	0.7	2.8
TC110393	Heavy metal domain containing protein	Metal ion transport GO:0030001	0.6	0.9	0.9^ns^	4.0
TC103767	Universal stress protein	Response to stress	1.1^ns^	0.8	1.4	3.1
TC95896	Dehydration responsive element binding	Regulation of transcription GO:0006355	1.6	ND	3.5	0.7
TC110815	AP2 domain transcription factor	Regulation of transcription	1.1	1.2	2.4	0.9^ns^
TC111267	Probable WRKY transcription factor 23	Regulation of transcription	1.5	1.3	2.4	1.4
TC102282	Probable WRKY transcription factor 28	Regulation of transcription	1.6	1.4	2.0	1.3^ns^
TC101761	Putative WRKY4 transcription factor	Regulation of transcription	1.3	1.0^ns^	3.2	1.2
TC97324	WRKY-type DNA binding protein	Regulation of transcription	1.8	1.3	3.5	0.9
TC103586	ZPT2	Regulation of transcription GO:0045449	2.9	ND	3.5	ND
						
	**Cell Death**					
TC103771	Ethylene up-regulated gene ER66	Ethylene signaling pathway GO:0009873	2.6	1.2	3.8	2.3
TC105302	Subtilisin-like proteinase	Proteolysis and peptidolysis GO:0006508	2.4	1.3	1.9	1.4
TC95356	probable serine proteinase	Proteolysis and peptidolysis	2.3	1.1^ns^	1.8	1.3
TC103618	Subtilisin-like protease	Proteolysis and peptidolysis	2.3	1.2	1.8	1.2
TC103261	Papain-like cysteine proteinase	Proteolysis and peptidolysis	2.8	0.9	4.7	ND
TC101194	T1N15.5	Apoptosis GO:0006915	2.0	1.1	1.5	0.9
TC112103	Subtilisin-type protease	Proteolysis and peptidolysis	1.8	0.9	1.1^ns^	2.3
TC107719	Putative cell death associated protein	Unknown	0.9	1.1	0.8	2.2
TC107153	Cystatin	Cysteine protease inhibitor GO:0004869	1.8	1.2	2.4	1.3
TC94966	Cystatin	Cysteine protease inhibitor	0.4	0.9^ns^	0.4	1.6
BI310700	Cystatin	Cysteine protease inhibitor	0.4	1.0	0.4	1.7
						
	**Senescence**					
TC101276	Rhodanese-like family protein	Aging GO:0007568	2.3	1.4	2.0	ND
TC101956	Senescence-associated protein sen1	Aging	2.1	0.8	5.2	1.3
TC107982	Probable senescence-related protein	Aging	1.2	ND	3.4	1.5
TC107460	Ntdin	Aging	1.4	1.1	2.1	1.3
TC94722	Putative senescence-associated protein	Aging	0.1	1.8	0.3	5.6
TC107766	SRG1 protein	Aging	0.8	0.4	0.8	0.8
						
	**Unknown/Miscellaneous**					
TC105342	MATE	Unknown	39.8	33.9	51.3	23.7
TC102211	GAST-1 protein precursor	Response to gibberellic acid stimulus	2.2	2.1	1.9	1.3
TC111698	COBRA-like gene	Unknown	16.3	1.7	18.8	ND
TC95697	F-box protein	Unknown	5.0	1.1^ns^	1.4	ND
TC108263	E3 ubiquitin ligase SCF complex	Protein catabolism GO:0006511	2.2	1.4	1.7	1.5

TIGR TC, The Institute for Genomic Research Tentative Consensus sequence ID version 8.0; Tentative Annotation, based on TIGR/NCBI tBLASTx tool; GO = Gene Ontology; Fold Change = ratio of transcript abundance in Al treatment/transcript abundance in control (-Al) treatment; ns = not significant; ND = not detected.

The Al-regulated expression changes in 12 significant genes were examined for both lines by quantitative reverse-transcription PCR (q-PCR). The q-PCR results showed the same direction of fold change of transcript abundance as the microarray in all three biological replicates for most genes (Table 3). In most cases, expression ratios based on q-PCR were higher than those ratios obtained from microarray hybridizations. This difference in expression ratios estimated by the two techniques has been observed previously [41] and reflects the specificity and sensitivity of the q-PCR technique [42]. The ratios (T32/S70) of the mean q-PCR fold change values were similar to the ratios of the microarray fold change values for most genes tested, thereby validating the microarray results.

**Table 3:** Real-time PCR validation of microarray results.

					qPCR fold change		Ratio (T32/S70)	
					
TIGR TC	Annotation	Time (h)	*M. truncatula *line	Microarray fold change	Mean	SE	Array	qPCR
TC105342	MATE	12	T32	39.8	44.0	7.8	0.8	0.7
			S70	51.3	67.4	6.4		
TC95697	F-box protein	12	T32	5.0	13.7	4.0	3.6	4.4
			S70	1.4	3.1	0.2		
TC103586	ZPT-2	12	T32	2.9	2.4	0.2	0.8	0.4
			S70	3.5	6.1	0.9		
TC111698	COBRA-like gene	12	T32	16.3	24.7	0.6	0.9	0.5
			S70	18.8	53.7	16.4		
TC93997	PR-protein PR-4	12	T32	2.6	2.5	0.3	0.5	0.5
			S70	5.0	5.0	1.1		
TC100486	Xyloglucan endotransglycosylase	12	T32	2.3	2.2	0.1	1.8	1.6
			S70	1.3	1.4	0.3		
TC96658	Xyloglucan endotransglycosylase	48	T32	2.9	3.7	1.0	0.3	0.1
			S70	10.7	38.3	5.6		
TC102211	GAST-1	48	T32	2.1	7.2	0.4	1.6	0.3
			S70	1.3	25.8	5.7		
TC111920	Polygalacturonase PG1 putative	48	T32	2.8	2.5	0.2	0.8	0.6
			S70	3.7	4.0	0.5		
TC103771	Ethylene up- regulated gene ER66	48	T32	1.2	1.3	0.2	0.5	0.3
			S70	2.3	4.6	0.8		
TC95154	Peroxidase	48	T32	3.9	1.2	0.1	1.3	0.4
			S70	3.0	2.9	0.1		
TC100155	Lipoxygenase	48	T32	0.5	0.4	0.0	0.7	0.6
			S70	0.7	0.6	0.1		

### Gene expression in response to 12 h of Al treatment in S70 root tips

**Figure 5:**
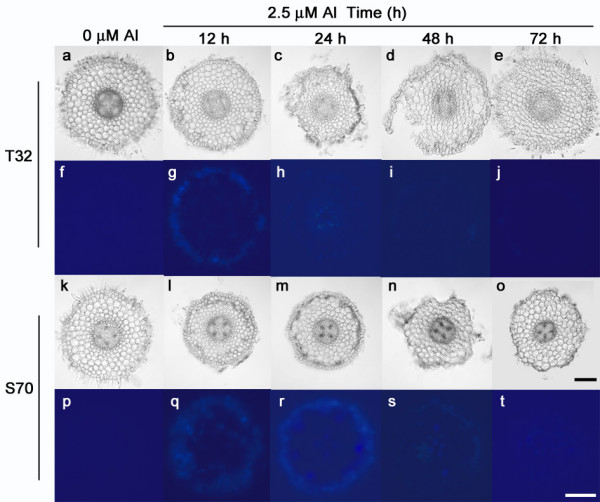
**Effect of Al on surface cell layer morphology and localization of Al by morin in transverse sections taken 1.5 to 2 cm behind root tips of *M. truncatula *lines.** Smooth surface of transversal sections of T32 (a) and S70 (k) roots grown in 0 μM Al solution. Recovery from Al-induced damage as a result of new root growth in T32 root apices (b-e) exposed to 2.5 μM Al for 12, 24, 48 and 72 h. Damaged outer epidermal and outer cortical cell layers as a result of no new root growth in S70 root apices (l-o) exposed to 2.5 μM Al. Black scale bar = 100 μm. Minimal background fluorescence observed in cells of T32 (f) and S70 (p) root apices grown in 0 μM Al solution. Bright blue fluorescence indicates morin staining in cells of T32 (g-j) and S70 (q-t) root apices grown in 2.5 μM Al solution for 12, 24, 48 and 72 h. White scale bar = 100 μm.

After 12 h of Al treatment, the RRG of S70 was approximately 45% (Figure 2b) with Al accumulating mainly in the epidermis and outer cortical cells of the root tip (Figure 5). Aluminum has been shown to rapidly bind to the pectic matrix in the cell wall, reduce cell wall (CW) extensibility [7,43], and consequently inhibit root elongation. Thus, as might be expected, a number of CW-related genes potentially involved in CW loosening including a pectinesterase precursor, an expansin-like protein and a xyloglucan endotransglycosylase, were differentially regulated in response to Al treatment in S70 root tips (Table 2). Pectin methylesterases have been shown to increase the Al sensitivity of plants because their ability to demethylate pectin, in addition to altering the sensitivity of the CW to the action of other CW-degrading enzymes [44], might create additional binding sites for Al in the CW [45,46]. This can occur by increasing the free organic acid moieties (in galacturnoic acid) in the wall, making it more negatively charged. Recently, we showed that the down-regulation of a pectin acetylesterase gene in *M. truncatula *transgenic roots resulted in a modest increase (~20%) in root growth under Al stress conditions compared to wild-type plants [31]. Based on these results it appears that plant cells respond to Al-induced CW stiffening by enhancing the expression of CW loosening enzymes in an attempt at stimulating CW expansion.

Notable at this time point was the accumulation of gene transcripts encoding reactive oxygen species (ROS) generating enzymes. Genes encoding peroxidases and peroxidase precursors as well as germin-like proteins and carbohydrate oxidase were up-regulated (Table 2). Aluminum toxicity has previously been shown to trigger the expression and activity of ROS generating enzymes and ROS accumulation has been shown to positively correlate with Al sensitivity [47,48]. ROS are capable of causing oxidative damage to proteins, DNA, and lipids in plant cells and may ultimately lead to cell death [49]. The enhanced expression of genes encoding ROS generating enzymes suggest that upon Al stress, S70 root tips experience oxidative damage, which could in turn result in complete root growth inhibition (Figure 2b). A number of genes coding for ROS generating enzymes were also found to be down-regulated in S70 at 12 h of Al treatment (Table 2). It is possible that specific peroxidases may be involved in the Al toxicity response, consequently resulting in the down-regulation of peroxidases involved in other responses, including ones that might be involved in ROS scavenging.

The observed increase in transcript accumulation of ROS generating genes may reflect ROS accumulation in root tips and therefore may elicit the expression of antioxidant-related genes. Consistent with this hypothesis, an increase in the expression of antioxidant genes was observed in S70 root tips. Genes putatively encoding glutathione S-transferase and thioredoxin were significantly up-regulated (Table 2). Also up-regulated were genes encoding tyrosine aminotransferase and 4-hydroxyphenylpyruvate dioxygenase, enzymes involved in the biosynthesis of α-tocopherols, which function as membrane stabilizers and antioxidants that scavenge oxygen free radicals, lipid peroxy radicals, and singlet oxygen [50].

A number of stresses induce production of ROS and lipid peroxidation, including pathogen attack. Since Al resulted in accumulation of ROS generating gene transcripts in S70 root tips, the resulting ions and oxidative damage might have triggered the up-regulation of a number of pathogen defense-related and membrane-stabilizing genes (Table 2). A number of genes encoding isoflavonoid biosynthetic enzymes were uniquely up-regulated in 12 h Al-treated S70 root tips (Table 2). The isoflavonoids, which are mostly limited to the subfamily Papilionoideae of the Fabaceae, have been shown to function as phytoalexins [51] and antioxidants [52]. Their up-regulation in response to Al stress may represent a unique response of the legume family to Al-induced oxidative stress.

**Figure 6:**
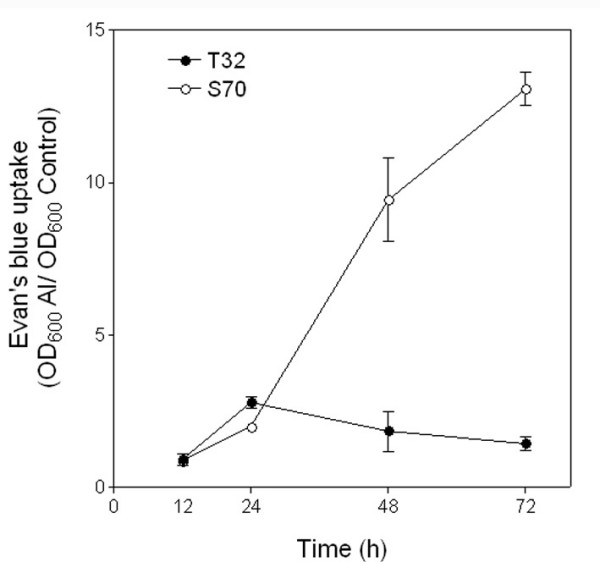
**Measurement of cell death by Evans blue uptake in *M. truncatula *root apices.** Relative Evans blue uptake (OD_600 _for Al treated root tips/OD_600 _for control (-Al) root tips) in root tips of *M. truncatula *lines T32 and S70 grown in 0 μM and 2.5 μM Al solutions for 12, 24, 48 and 72 h.

It has been shown that ROS triggers cell death by apoptosis, necrosis, or mechanisms with features of both [53]. Between 24 and 48 h of Al treatment, cell death of Al-accumulating epidermal and outer cortical cell layers was observed in S70 root tips. Fluorescence observed in root tip cells as a result of morin staining depicts Al-accumulation while both light microscopy of Al-treated root tip cross sections (Figure 5) and root tips stained with Evan's blue, which is a dye that measures extent of cell death, indicates cell death (Figure 6). Consistent with this phenotypic observation, a number of cell-death associated genes were up-regulated in S70 root tips including cysteine proteinase, senescence associated proteins and genes for CW degrading enzymes that may be necessary for cell separation during programmed cell death. Hypothetically, gene expression data from S70 root tips after 12 h of Al treatment reflects enhanced CW stiffening, severe oxidative damage probably due to significant ROS accumulation and activation of cell death.

### Gene expression in response to 48 h of Al treatment in S70 root tips

After 48 h of Al treatment, RRG of S70 was approximately 17% (Figure 2b). Notably, transcript accumulation of CW-modifying genes was observed at this time point (Table 2) suggesting continued CW stiffening from Al treatment. Furthermore, the prolonged induction of caffeic acid O-methyltransferase, involved in lignin biosynthesis only in S70 root tips could contribute to continued Al-induced root elongation inhibition. Genes putatively encoding polygalacturonase, pectate lyases, and β-1, 4-glucanase, which are CW-degrading enzymes, were significantly up-regulated in S70 root tips (Table 2). It is plausible that cell separation during the persistent cell death response at 48 h of Al treatment may elicit the prolonged expression of these genes. Interestingly, three genes encoding putative arabinogalactan-proteins (AGP) were uniquely up-regulated in S70 at 48 h of Al treatment. AGPs belong to a class of hydroxyproline-rich glycoproteins that are abundant in the plant CW and membrane [54], with no clear function. Two maize AGPs were found in disintegrating xylem cells and a role in identifying cells committed to PCD was proposed [55]. Furthermore, two AGP genes were identified as showing enhanced expression in Arabidopsis after Al stress [56]. Thus, AGPs may represent a novel facet of the Al toxicity response since they are significantly up-regulated only in the Al-sensitive line and may be involved in modulating cell wall architecture and/or involved in the Al-induced cell death response.

Accumulation of transcripts for enzymes generating ROS remained high after 48 h of Al treatment. The maintenance of peroxidase transcripts up to 48 h of Al treatment is consistent with previous observations in tobacco cells [57] and Arabidopsis [29] in which these genes showed enhanced expression during prolonged Al treatments. A few antioxidant genes including thioredoxin, ascorbate peroxidase, and alternate oxidase were uniquely up-regulated in S70 root tips at 48 h Al treatment. However, transcripts for other antioxidant related genes expressed after 12 h of Al treatment did not show enhanced accumulation at 48 h. The enhanced expression of a greater number of ROS generating genes compared to antioxidant genes at 48 h suggests that the antioxidant capacity of S70 root tips might be insufficient to prevent significant ROS accumulation. Consequently, higher doses of ROS without sufficient scavenging, may lead to greater oxidative damage and necrosis in S70 root tips. Consistent with this hypothesis, the transcripts of four PR-genes and other stress-related genes remained at elevated levels after 48 h of Al treatment suggesting continued oxidative damage. In addition, cell death and senescence-associated genes were uniquely up-regulated in 48 h Al-treated S70 root tips suggesting the longevity and severity of the cell death response. In agreement with the expression data, we observed a ≥ 10-fold extent of cell death in S70 Al-treated root tips compared to control root tips after 48 h of Al treatment (Figure 6). In addition, at these time points, Al was observed in some of the inner cortical layers of the root tip as indicated by morin staining (Figure 5).

Collectively, the prolonged expression of genes associated with CW loosening, ROS generation, cell death and CW degradation after 48 h of Al treatment implies that the Al-induced oxidative damage and cell death response resembles a necrotic response, damaging deeper cell layers and ultimately resulting in irreversible root growth inhibition in S70.

### Gene expression in response to 12 h Al treatment in T32 root tips

At 12 h of Al treatment, the RRG of T32 was approximately 78% (Figure 2a) with Al accumulating mainly in the epidermal cell layer of the root tip as indicated by morin staining (Figure 5). The inhibition in root growth, although not as severe as in S70, might induce the expression of CW-related genes. Consistent with this hypothesis, CW loosening genes were similarly regulated in S70 and T32 root tips at 12 h of Al treatment (Table 2), indicating an overlap in responses to Al toxicity in both lines.

Transcript accumulation of genes encoding ROS generating enzymes in T32 root tips was similar to that observed in S70; however, the total number of up-regulated genes was lower in T32 root tips (Figure 4). A number of antioxidant genes accumulated similarly in both lines (Table 2). However, two quinone oxidoreductase genes, a glutathione s-transferase (GST) gene, and a blue copper protein (BCB) precursor were significantly up-regulated only in T32 root tips. Quinone-oxidoreductases, which are involved in the detoxification of reactive aldehydes derived from lipid peroxides [58] have previously been shown to be induced in response to Al treatment in Al-tolerant and Al-sensitive rice roots [59]. Previously, BCB and GST genes were shown to be up-regulated in Arabidopsis in response to Al treatment [29] and plants over-expressing these genes displayed increased resistance to low Al concentrations [60]. Therefore, these genes may represent a resistance response associated with ROS scavenging in response to Al treatment only in Al-resistant root tips. Relative to S70, a greater number of ROS generating genes including peroxidases and peroxidase precursors were down-regulated in T32 (Table 2). However, in contrast to S70, antioxidant genes were not down-regulated in T32. In addition, up-regulation of fewer PR-genes, isoflavonoid biosynthetic genes, and stress-related genes (Table 2) reflected a lower extent of oxidative damage in T32 root tips. These data suggest that although there appears to be ROS accumulation in response to 12 h Al treatment in T32 root tips, the levels may be lower than that observed in S70.

A number of cell-death associated genes were uniquely up-regulated in T32 after 12 h of Al treatment (Table 2). Expression of these genes is consistent with cell death observed at 24 h of Al treatment (Figure 5 and 6). Interestingly, cell death appeared to be restricted to cells that accumulate Al as visualized by light microscopy of root tip cross sections (Figure 5). It has been suggested that low Al concentration treatments induce cell death possibly via a ROS-activated signal transduction pathway [61]. However, exposure to more toxic concentrations of Al may cause necrosis in the root tip cells. Likewise, H_2_O_2 _produced in barley roots, during early phases of Al stress, has been suggested to play a role in the induction of cell death [62]. Therefore, in addition to the observed patterns of expression of ROS generating and scavenging genes, the activation of cell death in Al-accumulating root tip cells indicate lower ROS accumulation in T32 after 12 h Al treatment. Accumulation of low levels of ROS, which although might be insufficient to cause significant oxidative damage, may play a key role in triggering cell death of Al-accumulating cells, as has been demonstrated in cell death responses to other abiotic and biotic stresses [49,63].

### Gene expression in response to 48 h Al treatment in T32 root tips

After 48 h of 2.5 μM Al treatment, the RRG of T32 recovered to approximately 90% (Figure 1 and 2a). At this time point fewer genes were differentially regulated in response to Al treatment in T32 root tips (Figure 4) compared to 12 h of treatment. Notably, fewer ROS generating genes were up-regulated, and up-regulation of a number of antioxidant genes was observed (Table 2). Since fewer ROS generating genes were up-regulated, the basal antioxidant capacity may have been sufficient to prevent significant ROS accumulation. Interestingly, there was no significant change in expression of stress-related genes, including PR-proteins and isoflavonoids and cell death genes (Table 2) suggesting that the ROS levels may have been insufficient to cause oxidative damage and cell death. In fact, down-regulation of some stress-related genes and senescence-associated genes was observed at this time point (Table 2). Moreover, probably as a result of new root tip growth, the extent of Al accumulation and cell death at 48 h was minimal (Figure 5 and 6). Therefore, we speculate that in T32 root tips, the cell death response occurred early on and was aimed at removing Al-accumulating cells leading to a recovery in root growth.

A continued response to Al stress in T32 root tips after 48 h of Al treatment was indicated by the high transcript accumulation of a putative multidrug and toxin extrusion (MATE) gene (TC105342; MtMATE). Although the expression of this gene was significantly up-regulated at both time points of Al treatment and in both lines (Table 2), the prolonged expression of this gene during root growth recovery in the Al-tolerant line made it an interesting candidate for Al resistance studies. We examined whether the spatial expression pattern of this gene played a role in differential Al resistance responses in these lines.

### Differential pattern of expression of MtMATE in T32 and S70

**Figure 7:**
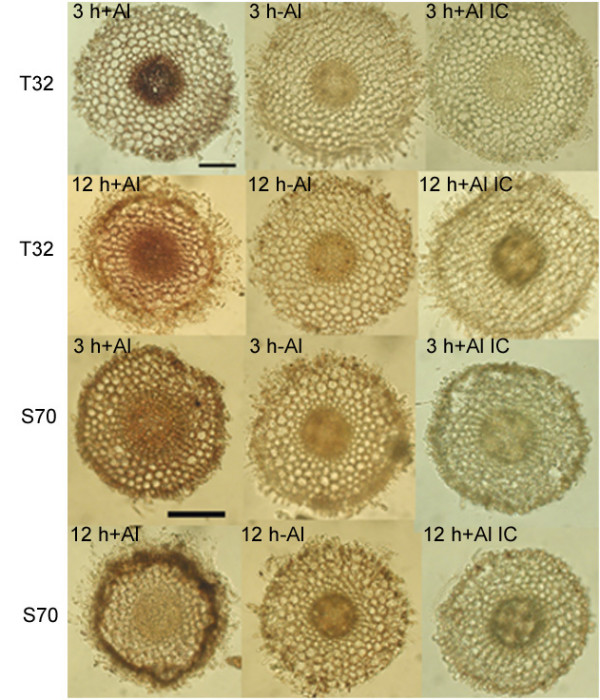
**MtMATE gene expression in Al-treated and control T32 and S70 root tips.** IC denotes *in situ *PCR using intron-specific primers. The images shown are representative of at least three independent experiments.

To determine the spatial distribution of MATE gene expression in response to early (3 h) and later (12 h) time points of Al treatment, *in situ *PCR was carried out on root tip cross sections. In 3 h Al-treated T32 root tips, a positive signal was visible in cells of the endodermis and the regions encompassing the vascular bundles (Figure 7). In contrast, in 3 h Al-treated S70 root tips, the signal appeared to be more uniformly distributed over the entire section including the epidermis, cortical cells and vasculature (Figure 7). No signal was observed in 3 h control (-Al) T32 and S70 root sections (Figure 7). In 12 h Al-treated T32 root tips, a strong signal was visible in the epidermis in addition to the vascular region; however, the signal appeared to be restricted to the epidermis and outer cortical cells in 12 h Al-treated S70 root tips. A positive signal was also observed in the epidermis, cortical cells and vasculature of 12 h control S70 roots (Figure 7) indicating the uniform expression of this gene under Al-free conditions. The intron-specific primers gave no positive signals (Figure 7). Overall, the localization of MATE expression in Al-treated root tips indicated that the transcript of the MATE gene accumulated most strongly in the vascular region of T32 and mainly in the epidermal and outer cortical layers in S70.

The MtMATE shares 67% identity with a putative lupin LaMATE (GenBank accession no. AAW30732; [64]), 63% identity with a rice MATE gene (GenBank accession no. ABB47036), and 62% identity with the Arabidopsis FRD3 protein (GenBank accession no. NP_187461.1; [65]). Based on studies conducted on different MATE genes it has been hypothesized that this family may be involved in transporting a diverse set of small organic molecules either directly out of the cell or into vacuolar compartments [65-67]. Recently, FRD3 was shown to be a citrate efflux transporter and Arabidopsis plants ectopically expressing FRD3 had significantly higher amounts of citrate in their root exudates compared to untransformed controls and possessed an enhanced resistance to aluminium [68]. Morin staining revealed presence of Al in the vasculature of T32 root tips at 12 h of Al treatment (Figure 5). Taking the differential root growth rates and Al accumulation patterns together with the spatial expression pattern of the MtMATE, it is tempting to speculate that the strong vascular expression of MtMATE in T32 root tips might be involved in transporting Al away from the sensitive growing root tip therefore resulting in lower root growth inhibition in T32. Internal detoxification of Al by formation of organic acid-Al complexes has been previously demonstrated in hydrangea and buckwheat [15,69]. In buckwheat, Al is chelated internally in the root cells by oxalate and translocated via the xylem to leaf cells where these complexes are then stored in vacuoles [70]. Likewise, it is possible that in T32 the MtMATE gene may be involved in the transport of organic acid-Al complexes into the aerial parts of the plant followed by sequestration into vacuoles. In S70, the organic acid efflux or Al-sequestration into vacuoles might be restricted to the root surface as indicated by the strong expression in the epidermal and cortical cells. In addition, it is possible that the observed expression of this gene under Al-free conditions in S70 root tips is in response to low pH or low pH-induced nutrient deficiencies. Interestingly, the LaMATE displays enhanced expression under various nutrient stress conditions including -P, -Fe, -Mn, -N and + Al [64]. Similarly, the FRD3 gene is induced in response to cold stress and during senescence (a search of 2,620 ATH1 Affymetrix chips in ). Alternatively, although Al-induced expression of the MATE gene occurs in both lines, the subsequent activation of organic acid efflux might be delayed or inhibited in S70. Localization of MtMATE to either the plasma membrane or the vacuole would reveal its site of action, and identification of the molecule it transports could permit a direct biochemical test of its function in transport.

## Conclusion

One of the most widely studied mechanisms of Al resistance is the Al exclusion and/or internal detoxification via organic acids (OA), which serve as Al chelators. However, induction of OA biosynthetic genes was not observed in this study. This finding was not surprising since, to date, no strong evidence exists for Al-induced expression of any of the enzymes catalyzing OA synthesis and metabolism [6]. Indeed, it is possible that OA biosynthetic enzymes are regulated translationally or post-translationally. In the present study we compared physiological and molecular differences between an Al- resistant and sensitive line, which enabled us to identify novel facets of Al resistance in *Medicago truncatula*. A number of Al-inducible genes with potential roles in Al resistance were identified in this study. For example, the Al-inducible MATE gene might be involved in organic acid exudation and internal detoxification of Al in T32, supporting the idea of an Al resistance mechanism involving organic acids in *M. truncatula*. Additionally, cell death of Al-accumulating cells in T32 represents a unique aspect of Al resistance in legumes. Our results provide a valuable data set for future studies targeted at investigating additional Al resistance responses.

## Methods

### Plant material

Seeds of 46 accessions of *Medicago truncatula *Gaertn. were obtained from the USDA-Western Regional Plant Introduction Station at Pullman, Washington, and seeds for another eight accessions (inbred lines) were obtained from INP-ENSAT, Toulouse, France. The selected accessions represent collections from different geographical locations and areas where acid soils are known to occur. To develop inbred lines from the USDA collection, one seed from each accession was grown in a greenhouse and self-pollinated. Seeds from these lines were used for subsequent experiments.

### Solution culture experiments

Six *M. truncatula *seeds from each line were scarified using concentrated sulfuric acid and surface sterilized with 5% (v/v) household beach for 3 min. The seeds were placed at 4°C for 2 d and germinated overnight on 1% agar plates in the dark at room temperature. Seedlings with radicles of about 1 cm in length were sown through the mesh bottoms of polypropylene cups. The cups were placed in precut holes of a plastic insert placed over a plastic tub that held 7.3 L of aerated nutrient solution. The nutrient solution contained: 1.2 mM KNO_3, _0.8 mM Ca(NO_3_)_2_, 0.1 mM NH_4_H_2_PO_4_, 0.2 mM MgSO_4_, 10 μM FeNaEDTA, 50 μM KCl, 12.5 μM H_3_BO_3_, 1 μM MnSO_4_, 1 μM ZnSO_4_, 0.5 μM CuSO_4_, 0.1 μM Na_2_MoO_4_, and 0.1 μM NiCl_2 _[71]. The pH of the nutrient solution was adjusted to 4.5 using 1 N HCl. Seedlings were grown for 72 h in a growth chamber (light/dark, 14/10 h) under a light intensity of 440 μmol photons m^-2 ^s^-1^. Al treatment was initiated after 72 h by replacing the control growth solution with an identical solution that contained 2.5 μM Al added as AlK(SO_4_)_3_. The control and treatment solutions were adjusted to pH 4.5. Root growth measurements were made at 0 and 48 h following Al exposure. Relative root growth was calculated as: RRG (%) = (root growth in Al solution/root growth in control solution) × 100. The RRG value of each line was normalized to that of *M. truncatula *Jemalong A17 to account for any variation in solution pH or composition in the different tubs.

For the dose-response experiments, plants were grown in 0 (control), 1.25, 2.5, 5.0 and 10 μM Al solutions, and root growth measurements were made at 48 h following Al exposure. For the time-course experiments, root growth measurements were made at 0, 12, 24, 48 and 72 h in 0 μM (control) and 2.5 μM Al solutions. These experiments were performed in triplicate.

### Hematoxylin and morin staining

For hematoxylin staining, control and Al treated roots were rinsed for 30 min in deionized water, with the water replaced twice during rinsing. Roots were then stained with hematoxylin (0.1% w/v hematoxylin, 0.01% w/v KIO_3_) for 30 min, and subsequently washed with deionized water for 30 min. For morin staining, control and Al treated roots were washed in MES buffer, pH 5.5, for 10 min, and stained with 100 μM morin in the same buffer for 1 h, as described previously [72]. Morin fluorescence was visualized using an Olympus inverted microscope (IX70, Olympus, NY) with fluorescence attachments.

### Evans blue quantification

Evans blue is a stain that indicates the loss of plasma membrane integrity of cells. Control and Al treated roots were rinsed with deionized water and stained with Evans blue solution (0.025% [w/v] Evans blue in 100 μM CaCl_2_, pH 5.6) for 10 min. The stained roots were washed three times with 100 μM CaCl_2 _(pH 5.6), until dye no longer eluted from the roots. Evans blue stain retained by cells was quantified as described [73] with minor modifications

### Plant growth conditions, Al treatment and tissue collection for microarrays

For the microarray experiment, T32 and S70 seedlings were grown in solution culture as described above. Root tips (approximately 0.5 cm in length) from control and 2.5 μM Al treated seedlings were harvested at 12 and 48 h after Al treatment and immediately frozen in liquid nitrogen. Samples were collected from three independent biological replicates and stored at -80°C. To verify phenotype of roots harvested for RNA isolation, root growth measurements of 12 sample roots were made at 0, 12 and 48 h following Al exposure.

### Microarray hybridization and analysis

Total RNA from control and Al-treated root tips was extracted using the RNeasy Plant Mini Kit (Qiagen, Alameda, CA) following the manufacturer's protocol. RNA was quantified spectrophotometrically and stored at -80°C before use. cDNA synthesis was carried out using a 3DNA Array 900 Expression Array Detection Kit (Genisphere, Inc., Hatsfield, PA) according to the manufacturer's instructions (3DNA Array 900 Expression Array Detection Kit, Appendix A, Genisphere).

The *Medicago truncatula *AROS (version 1.0) arrays (Operon Biotechnologies Inc., Huntsville, AL) containing 16,086 70 mer probes representing 16,086 *M. truncatula *genes were used for the microarray studies. The 70 mer probes were printed on Telechem Super Amine slides (Sunnyvale, CA) with spot size approximately 100 to 110 μm in diameter. Slides were processed prior to use by rehydrating over a 50° to 55°C water bath for 5 to 10 sec and snap-drying on a 65°C heating block for 5 sec, approximately three to four times. The DNA on the slide was cross linked by exposing the DNA-side-up to 65 mJ in a UV crosslinker. The slides were washed in 1% SDS at room temperature for 5 min, dipped in 100% ethanol for 30 sec with gentle agitation, centrifuged at 1,500 rpm for 2 to 5 min and stored in a light-proof box under cool and dry conditions before use. Microarrays for each time point were hybridized to cDNAs from both Al-treated and control roots, with cDNAs from the two different treatments labeled with Cy5 and Cy3 dyes. Each hybridization was repeated at least six times to account for technical variability, with triplicates of each dye combination to control for dye effects. A modified two-step hybridization reaction was performed as described in the 3DNA Array 900 Expression Array Detection Kit (Genisphere). Slide scanning and data analysis was performed as described in [74].

### Quantitative reverse transcription PCR

Root tip RNA samples from the three biological replicates that were used for microarray experiments were used for quantitative real-time PCR (q-PCR) assays. Primers for q-PCR reactions were designed using the Primer Express software (v 2.0, Applied Biosystems, Foster City, CA) and are presented in Table 4. RNA extraction and PCR conditions are as described previously [75]. For each biological replicate, three q-PCR reactions were run from a cDNA synthesis and the mean values presented. Amplification of 18S ribosomal RNA was used as the endogenous control. The ΔΔCt (threshold cycle) method was used to calculate relative fold changes between Al-treated and control (-Al) cDNA samples. Specificity of the product was confirmed by a single peak in a dissociation curve at the end of the PCR reaction.

**Table 4:** Primer sequences used for quantitative RT-PCR.

TIGR TC	Forward Primer	Reverse Primer
TC105342	5'-CATCCCTCTCTTGCACCATCA-3'	5'-TTTCCACTTCTTGTTGGGTTCA-3'
TC102211	5'-TCCAGCTCCACAACCTAGCA-3'	5'-TGCATCGTGGTCCACATTCT-3'
TC95697	5'-GGTGTCGAAGGTGGCCATAG-3'	5'-TCAGCACGACCGTAAAATCTTG-3'
TC100486	5'-CATCTGGAAGCCACAACACATTA-3'	5'-GTTGGTTCTTTGGGAATGGAATAC-3'
TC11192	5'-AGTGCAACTGTTGCTTGCACAA-3'	5'-CCATAAGCATTCCAACAAAAAGG-3'
TC96658	5'-GCTGAGGTGGGTGCAGAAGA-3'	5'-GAGACTAAGAGTGAGTGCATTCAACTG-3'
TC93997	5'-GTGGTCAAGCTTCTTGTGGAAAG-3'	5'-CAACCCTCCATTGCTGCATT-3'
TC103771	5'-TGGCAGGGAGAGGACAGTTG-3'	5'-CGGCTGGTGTTCTACCAGAAG-3'
TC95154	5'-GTAGCTCTGTCAGGAGGGCATAC-3'	5'-AACGAAGGGTCCACGTCATG-3'
TC100155	5'-TTGAGATTTCTTCTGCAGTTTACAAGA-3'	5'-GGAAGAGGGATCCTCAACAGCCTA-3'
TC101956	5'-ACCTCATAGTGGGTTGCCAAA-3'	5'-ATAACCTCCTCCCATGTTGTACACA-3'
TC103586	5'-TGAATGCAAAACGTGCAACA-3'	5'-TGAGTTCTTCACCTTCAGCTAGTTTC-3'
18s rDNA	5'-CCTCAAACTTCCGTGGCCTAA-3'	5'-TAACGAACGAGACCTCAGCCTG-3'

### In-well *in situ *PCR on root tip sections

T32 and S70 seedlings were grown in solution culture as described for the microarray tissue collection and root tips (approximately 0.5 cm in length) were harvested from control (0 μM) and Al (2.5 μM) treated seedlings at 3 and 12 h following Al exposure. *In situ *PCR on root tissue was carried out as described [76] with minor modifications. The root tips were fixed in 4% paraformaldehyde, 0.25% glutaraldehyde, 0.003 N sodium hydroxide, and 1% Tween 20 in 100 mM PIPES buffer (pH 6.8). The MATE exon primer for the reverse transcription reaction was 5'-AGCAATGGAAACTCCAGCAGC-3' and the primers for the PCR reaction were MATE exon forward, 5'-CATCCCTCTCTTGCACCATCA-3'or MATE intron forward, 5'-GCAAAGAGGAACAATGGCGA-3' and MATE exon reverse, 5'-AGCAATGGAAACTCCAGCAGC-3'. The sections were examined under a microscope and photographed with a digital camera (Nikon Coolpix 900).

## Authors' contributions

DC and DAS conceived the study and wrote most of the paper. DC and DFG conceived the physiological characterization of the Al resistant and sensitive lines, while DC and DAS conceived the molecular characterization of the two lines. NS performed the statistical analyses of the microarray data and uploaded the data onto Gene Omnibus. KAV improved the overall quality of the manuscript. All authors read and approved the final manuscript.
